# Exploring the Use of Ambientally Stored Methylene Diphosphonate Radiopharmaceutical Aliquots in Solving Challenging Situations in Developing Countries

**DOI:** 10.1055/s-0044-1788278

**Published:** 2024-07-04

**Authors:** Jenipher None Zulu, Reuben None Katebe, Martalena None Ramli, Rita None Sakala, Elias None Mwape, Ernest None Chipasha, Bernard Mudenda Hang'ombe

**Affiliations:** 1 Pharmacy/School of Health Sciences, Levy Mwanawasa Medical University, Zambia; 2 Department of Technology and Science, Ministry of Higher Education, Lusaka, Zambia; 3 Department of Nuclear Science, National Institute for Scientific Research, Lusaka, Zambia; 4 Department of Radiopharmaceuticals, Centre for Radiopharmaceutical Production, Indonesia; 5 Department of Nuclear Medicine/Radiopharmacy Unity, University Teaching Hospital, Zambia; 6 Department of Nuclear Medicine, University Teaching Hospital, Zambia; 7 Office of the Deputy Commandant, Northern Command Military Hospital, Zambia; 8 School of Veterinary Medicine, University of Zambia, Zambia

**Keywords:** radiopharmaceutical, technetium, methylene diphosphate, ambient conditions, radiochemical purity

## Abstract

**Objectives** The primary aim was to evaluate the prolonged quality characteristics of methyl diphosphonate (MDP) aliquots during ambient storage over a specified duration. This study further investigated potential additives that could enhance the stability of MDP aliquots stored under such conditions.

**Materials and Methods** This was a laboratory-based experimental study conducted at the University Teaching Adult Hospital in Lusaka, Zambia. A total of 36 MDP aliquots stored at ambient conditions and 4 MDP aliquots stored at conventional refrigerated frozen conditions were labeled with technitium-99m ( ^99m^ Tc) and tested for radiochemical purity (RCP) and other quality characteristics. A comparative analysis of the stability and quality of MDP aliquots from the two cohorts was then conducted.

**Statistical Analysis** Stata 14 was used to analyze the data on the RCP of all MDP aliquots.

**Results** The RCP of ambient stored MDP aliquots was found to be ranging from 98 to 99%, while that for frozen and refrigerated ones ranged from 99 to 100%. There was also a 1% increase in RCP for both cohorts with argon gas purging (98 and 99%, respectively).

**Conclusion** The RCP of MDP aliquots from both cohorts was much higher than the required minimum of 90% implying that there was no significant association of their stability and quality with the mode of storage. However, purging with argon gas seemed to increase the stability further in both streams. The study findings show potential for application in resource-constrained environments and centers, especially in developing countries, where challenges to maintain the cold storage chain of these important radiopharmaceuticals are likely to be encountered due to power outages.

## Introduction

Radiopharmaceuticals (RPs), consisting of a radioactive isotope combined with a pharmaceutical compound, offer exceptional precision when it comes to diagnosing and treating various medical conditions. 1 RPs have revolutionized the field of medicine, providing precise diagnostic capabilities and innovative treatments for a wide range of ailments. 2 3 RPs are, therefore, widely used in diagnostic imaging procedures like single-photon emission computed tomography and positron emission tomography. 4 5 6 7 The precision of these imaging techniques allows healthcare professionals to identify and evaluate various ailments with accuracy. 8

However, while RPs offer remarkable accuracy in diagnosis and treatment, several usage challenges exist. 9 These include the short half-lives of certain radioisotopes, limited availability, and cost considerations. 6 Other challenges come in when there are low patient volumes and therefore a whole multidose vial of a RP kit that can cater for a good number of patients tends to go to waste.

Further, though the use of RPs is integral for diagnostic and therapeutic applications, 10 11 12 13 logistical challenges associated with their storage and transportation, particularly in resource-constrained environments of developing countries, often hinder the seamless provision of nuclear medicine services. However, the compound methylene diphosphonate (MDP—supplied by AEC Armersham, 6 Indianapolis ST, Kyalami Park, Midland, 1684, South Africa), a RP commonly employed in bone imaging, holds promise for mitigating these challenges. While literature holds that MDP aliquots stored at refrigerated and frozen conditions remain viable for as long as 9 months, 14 15 the effects of ambient conditions on aliquots in areas with unreliable cold chain backup strategies have not been studied and, therefore, the viability of MDP aliquots kept at ambient conditions has not been explored. This research, therefore, investigated the potential of ambient-stored MDP RPs to overcome the hurdles associated with cold-chain maintenance and storage infrastructure limitations.

It is hoped that the study findings would contribute valuable insights that could pave way to a more sustainable and accessible deployment of nuclear medicine technologies, 16 17 18 19 thereby improving diagnostic capabilities and patient outcomes in resource-challenged regions.

## Materials and Methods

### Preparation of Various Types of Normal Saline

Two streams of different types of normal saline (NS) were prepared using two 500 mL packets of NS. The first packet (named as NS stream) was left unchanged, while the other 500 mL packet (named as NSA stream) was purged with argon gas.

### Preparation of MDP Aliquots

MDP aliquots were prepared by diluting a vial of MDP RP kit with 4 mL of NS drawn from either stream (NS and NSA). Thereafter, 1 mL was withdrawn from each diluted MDP vial and dispensed into four separate sterile vials. This process was done on ten vials of MDP RP kit making each stream of NS yield 20 MDP aliquots.

From each stream of NS, 18 MDP aliquots were stored under ambient conditions (defined as the room temperature that existed within our laboratory during the research period and which ranged from 25°C to 35°C) until the 18th day, while one MDP aliquot was stored refrigerated, and another one was kept frozen for a duration of 28 days.

### Radiochemical Purity Test

Radiochemical purity (RCP) testing was conducted daily on a total of 36 MDP aliquots stored under ambient conditions ranging from 1 to 18 days. Additionally, testing was performed on a total of four 13 MDP aliquots stored under refrigerated and frozen conditions on the 28 day. This RCP test involved labeling the aliquots with 600 to 1,200 MBq of technetium solution following a fresh elution. 20

This was done by dropping 0.05 mL of technetium-labeled MDP aliquots applied to two Whatman chromatographic paper strips. These were then submerged in two distinct mobile phases (NS and methyl ethyl ketone [MEK]) to determine the concentrations of radiochemical impurities and expected form of the radiochemical compound. Free technetium pertechnetate ( ^99m^ TcO ^4−^ ) and the colloid also known as hydrolyzed reduced pertechnetate ( ^99m^ TcO11) in 99 ^m^ Tc labeled complexes were found as radiochemical impurities. Poor-quality pictures and an extra dosage of radiation for the patient come from the presence of high level of radiochemical contaminants in a RP.

The Whatman paper was employed as the immobile phase to detect free ^99m^ TcO ^4−^ , while MEK and NS were utilized as the mobile phase. Free ^99m^ TcO ^4−^ is soluble in both NS and MEK; as a result, it migrated with the mobile phase as the paper got wet until the expected mark and the reference values were noted. Whatman paper number 1 served as an immobile phase and MEK served as a mobile phase to determine the colloid, ^99m^ TcO11. Sodium chloride is insoluble in the colloid ( ^99m^ TcO11) and required RP. To do this, a strip of 0.9 cm × 8.5 cm Whatman paper was cut, marked with a pencil to indicate the origin at 1 cm from the bottom, and marked with a pencil to indicate the solvent front line at 6.5 cm from the origin.

The two chromatography paper strips were immediately put in the developing chamber for chromatography with a lid containing MEK and NS and allowed to develop once a drop (0.05 mL) of the mixture was detected on the origin line. The strip was removed and left to dry when the solvent got to the solvent front line. The amount of ^99m^ Tc-MDP, free ^99m^ TcO ^4−^ , and ^99m^ TcO11 colloid were typically measured on chromatograms made from RCP using the dosage calibrator. According to the World Health Organization (WHO), ^99m^ Tc-MDP should only be used if the RCP is at least 90%.

## Results

**Fig. 1:**
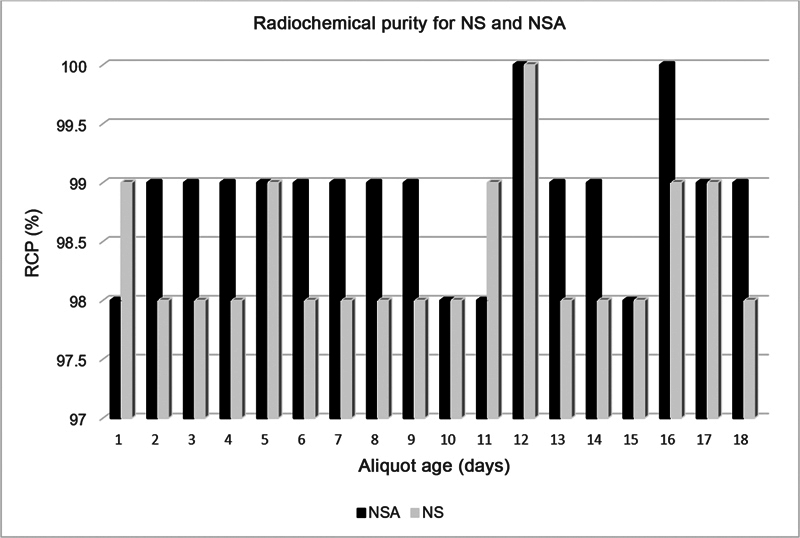
Radiochemical purity (RCP) of ^99m^ Tc-methylene diphosphonate aliquots reconstituted with normal saline aliquot (NSA) and with normal saline (NS), only those stored at ambient conditions. The NSA stream is represented by the black color, whereas the NS stream is represented by the color gray.

The MDP was reconstituted according to the manufacturer's instructions and then subjected to different storage temperatures and time period. As indicated in Table 1 , the storage time ranged from 18 to 28 days at room temperature ranging from 25 to 42°C for ambient stored MDP aliquots and from 2 to -4°C for MDP aliquots stored at recommended conditions. The RCP that was our dependent variable showed a reduction of 1 or 2% from the one stored at recommended conditions. The reduction, however, passed the WHO standards. The RCP findings comparison between the NSA and NS stream is shown in Fig. 1 .

**Table 1:** RCP of ^99m^ Tc-MDP aliquots made from the two streams of NS stored at ambient conditions (NS and NSA) and those kept at recommended conditions

Aliquot stream	No of aliquots	Aliquot age (days)	Storage temp. (°C)	RCP (%)	WHO (>90)
NS	1	28	−23 (frozen)	100	Passed
	1	28	3 (refrigerated)	99	Passed
	18	18	27 (ambient)	98	Passed
NSA	1	28	−23 (frozen)	100	Passed
	1	28	2 (refrigerated)	99	Passed
	18	18	27 (ambient)	99	Passed

Abbreviations: NSA, normal saline aliquot; RCP, radiochemical purity; ^99m^ Tc-methylene diphosphonate; WHO, World Health Organization.

## Discussion

The goals of this research were to determine the effect of storage conditions and additives on the RCP. This section discusses the outcomes that were achieved.

### Radiochemical Purity (RCP) of Aliquots of 99 ^m^ Tc-MDP

Table 1 provides the RCP findings of 99 ^m^ Tc-MDP aliquots held at various settings. Both streams of normal saline (NS and NSA) surpassed the WHO's recommended criterion of at least 90% for RCP, despite differences in storage duration and temperature. The small but statistically significant decrease is RCP seen across all examined time periods suggests stability as seen in Table 2 .

**Table 2:** Statistical inference of NS and NSA streams of MDP aliquots

	Argon purged NSA					
Normal Saline (NS) RCP (%)						Total
	0.98	3 0.1	9 0.3	0 1.3	0 0.6	12 2.3
	0.99	1 0.0	3 0.0	1 0.4	0 0.3	5 0.7
	1.00	0 0.2	0 0.6	1 7.6	0 0.1	1 8.5
	NS RCP	0 0.2	0 0.6	0 0.1	1 17.1	1 18.0
	Total	4 0.5	12 1.5	2 9.4	1 18.0	19 29.4
		** Pearson chi ^2^ (9) = 29.4500 **		**Pr = 0.001**		

Abbreviations: MDP, methyl diphosphonate; NSA, normal saline aliquot; RCP, radiochemical purity.

**Fig. 2:**
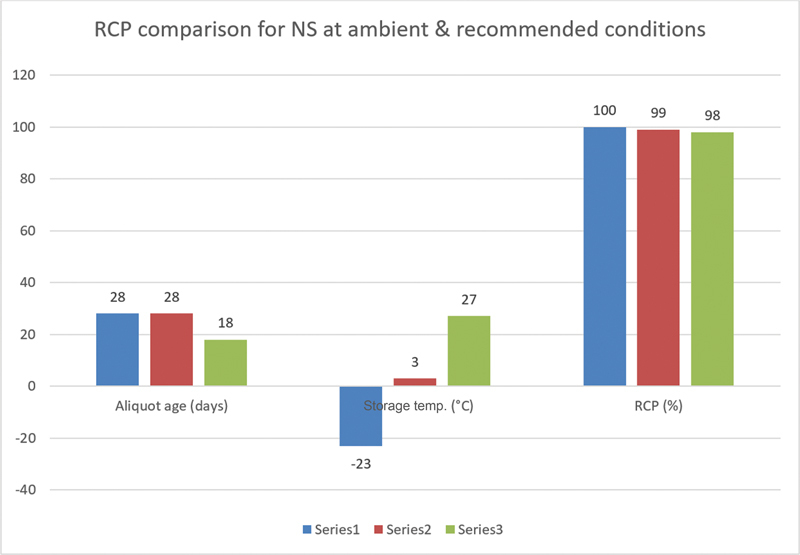
Radiochemical purity (RCP) comparison for ^99m^ Tc-methylene diphosphonate aliquots made from the normal saline (NS) stream under different storage conditions: Series 1 (−23°C), Series 2 (2–8°C), and Series 3 (ambient conditions, 25–35°C).

**Fig. 3:**
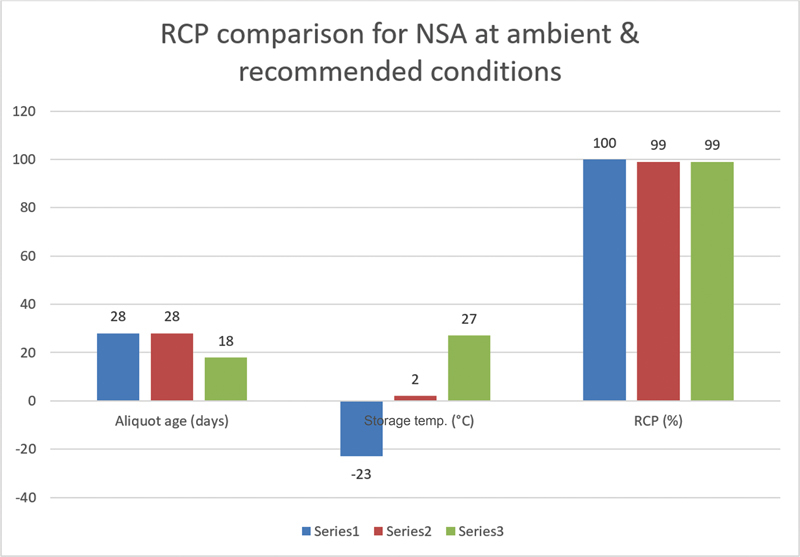
Radiochemical purity (RCP) comparison for ^99m^ Tc-methylene diphosphonate aliquots made from normal saline aliquot (NSA) and stored under various conditions: Series 1 (−23°C), Series 2 (2–8°C), and Series 3 (ambient, 25–35°C).

Fig. 1 clearly exhibits the comparison of RCP results between different streams, illustrating the constancy in RCP throughout the streams. Figs. 2 and 3 indicate a comparison of NS and NSA MDP aliquots with those stored at recommended conditions. The findings from this research revealed that MDP aliquots stored at room temperature remained viable for almost a month with RCP of 98 to 100%. Thus, MDP aliquots stored at ambient temperatures have acceptable quality control results and may be used to diagnose cancer and infections, especially in low-resource countries with cold chain concerns.

Thokchom et al studied how MDP kit fractionation affects RP RCP and biodistribution using vial and syringe storage methods. The aliquots were kept at −20°C. In their study, the efficacy and RCP of ^99m^ Tc-MDP were compared with those of a traditional process without fractionation. 20 Fractionated ^99m^ Tc-MDP in the vial and syringe had more than 95% RCP although by day 8, RCP had dropped to 83.6 and 88%, respectively. Biodistribution studies were assessed in a cohort of 100 patients. Dhingra and colleagues evaluated the Radiochemical Purity (RCP) of MDP, Di Mercapto Succinic Acid (DMSA), and Diethylenetriamine Penta Acetate (DTPA) kits, with average RCP values of 95%, 91%, and 95%, respectively. 21 They examined 90 samples altogether (30 of each) after adding NS and refrigerating the aliquots in evacuated vials at −20 to −30°C for 1 to 15 days. Bayomy et al studied Egyptian approaches to lower MDP kit prices. After 30 days, the RCP was tested upon which it was discovered that the aliquots were effectively maintained with an average RCP of 98.56% for fractions maintained between −20 and −28°C in nonoxygenated vials and 97.15% for fractions maintained between 0 and +4°C. 22 Successful biodistribution studies localized in the region of interest were seen in the human subjects.

### Consequences and Prospects for the Future

Ambient stored aliquots of ^99m^ Tc-MDP RP were studied for their stability and behavior under various storage circumstances. The stable RCP concentrations provide evidence for the stability of the RP product. To better understand how ambient stored MDP aliquots, researchers should dig further into the particular processes patterning to their biodistribution patterns as this will also help generalize and apply the findings to a wider context.

## Conclusion

In conclusion, this study successfully investigated the stability of ambient stored MDP RP aliquots. The RCP results demonstrated the resilience of MDP aliquots, with both streams meeting the WHO's recommended threshold of at least 90% RCP. Despite minor reductions in RCP levels, well within an acceptable margin, the RP maintained its integrity over the tested duration and temperature ranges.

The statistical analysis using Stata version 14 added robustness to the findings, ensuring a reliable interpretation of the data. This study adhered to ethical standards and obtained necessary approvals, further validating the credibility of the results.

These findings contribute to the understanding of ambient stored MDP aliquots behavior, laying the groundwork for potential further future research.

As a concluding remark, this research not only provides insights into the specific context of 99 ^m^ Tc-MDP but also sets the stage for further investigations into the broader implications of storage conditions and additives on RP performance, ultimately advancing the field of nuclear medicine.

This study evaluated the use of ambiently stored MDP aliquots, a radiopharmaceutical employed in diagnosing cancer and infectious diseases. Future research could compare the cost-effectiveness of using fractionated versus unfractionated MDP. Additionally, further studies could investigate the stability of ambiently stored MDP aliquots over periods longer than eighteen days, particularly in hotter regions of Zambia.

## References

[1] Weber W A Johannes C Carolyn J A The future of nuclear medicine, molecular imaging, and theranostics J Nucl Med 2020 2 61 263S 272S 10.2967/jnumed.120.254532 33293447

[2] Vallabhajosula S Berna D P John W B Molecular imaging of prostate cancer: radiopharmaceuticals for positron emission tomography (PET) and single-photon emission computed tomography (SPECT) Precision molecular pathology of prostate cancer 2018 475 501

[3] Dougherty D D Rauch S L Fischman A J Positron emission tomography and single photon emission computed tomography Essentials Neuroimaging Clin Pract 2004 13 10 75 82

[4] St James S Bednarz B Benedict S Current status of radiopharmaceutical therapy Int J Radiat Oncol Biol Phys 2021 109 04 891 901 32805300 10.1016/j.ijrobp.2020.08.035 PMC8422258

[5] Bombardieri E Bonadonna G Gianni L Breast cancer: nuclear medicine in diagnosis and therapeutic optionsSpringer Science & Business Media;2007Dec 22

[6] Shende P Gandhi S Current strategies of radiopharmaceuticals in theranostic applications J Drug Deliv Sci Technol 2021 64 102594

[7] Urbain J L Scott A M Lee S T Theranostic radiopharmaceuticals: a universal challenging educational paradigm in nuclear medicine J Nucl Med 2023 64 06 986 991 37142302 10.2967/jnumed.123.265603

[8] Assaad T New amino bisphosphonate compound for skeletal imaging: Comparison study with methylenediphosphonic acid (MDP) and (1-hydroxyethane-1, 1-diyl) diphosphonic acid (HEDP) Nukleonika 2016 61 01 69 74

[9] Chopra A 99mTc-Methyl diphosphonate Mol Imaging Contrast Agent Database 2009 24 1(MICAD)

[10] Schillaci O Filippi L Manni C Santoni R Single-photon emission computed tomography/computed tomography in brain tumors. In Seminars in nuclear medicine WB Saunders 2007 01 37 34 47 10.1053/j.semnuclmed.2006.08.003 17161038

[11] Lange R Schreuder N Hendrikse H Radiopharmaceuticals: Practical Pharmaceutics: An International Guideline for the Preparation, Care and Use of Medicinal Products Cham Springer International Publishing 2023 531 550

[12] Okarvi S M Recent developments of prostate-specific membrane antigen (PSMA)-specific radiopharmaceuticals for precise imaging and therapy of prostate cancer: an overview Clin Transl Imaging 2019 7 189 208

[13] Vallabhajosula S Lilja S Brigitte V A broad overview of positron emission tomography radiopharmaceuticals and clinical applications: what is new?. Seminars in nuclear medicine WB Saunders 2011 4 41 246 264 10.1053/j.semnuclmed.2011.02.003 21624560

[14] Bayomy T Abdulrazzak M Moustafa H Khalil W M Pant G S Radiopharmaceutical, original article different models for cost-effective fractionation of bone radiopharmaceuticals; methylene diphosphonate (MDP) & hydroxy methylene diphosphonate (HDP) Egypt J Nucl Med 2009 2 02 74

[15] Fleming W K Jay M Ryo U Y Reconstitution and fractionation of radiopharmaceutical kits J Nucl Med 1992 33 10 1915 1403166

[16] Negi M Dhingra M Dhingra V K Quality of radiochemical purity in multiple samples of various fractionated cold kits: testing a cost & time effective technique Hell J Nucl Med 2019 22 03 200 205 31587030 10.1967/s002449911056

[17] Bhatia N Dhingra V K Watts A Fractionation of common cold kits as a cost-saving method in a low-volume nuclear medicine department Indian J Nucl Med 2013 3 28 S36

[18] Penglis S Tsopelas C 99Tcm-tetrofosmin: evaluation of fractionated cold kits and two new methods of quality control Nucl Med Commun 2000 21 05 469 472 10874705 10.1097/00006231-200005000-00010

[19] Bayomy T Abdulrazzak M Moustafa H Khalil W M Pant G S Radiopharmaceutical, original article different models for cost-effective fractionation of bone radiopharmaceuticals; methylene diphosphonate (MDP) and hydroxymethylene diphosphonate (HDP) Egypt J Nucl Med 2009 2 74

[20] Thokchom A K Kumar R Bharati S Quality assessment of Tc-99m methylene diphosphonate (MDP) radiopharmaceutical prepared using different cold kit fractionation methods Indian J Nucl Med 2022 37 01 7 11 35478684 10.4103/ijnm.ijnm_115_21 PMC9037872

[21] Decristoforo C Siller R Chen F Riccabona G Radiochemical purity of routinely prepared 99Tcm radiopharmaceuticals: a retrospective study Nucl Med Commun 2000 21 04 349 354 10845223 10.1097/00006231-200004000-00009

[22] Waight L A Cunnane C M O'Brien L M Millar A M Effect of 99Tc on the radiochemical purity of 99mTc radiopharmaceuticals Nucl Med Commun 2011 32 08 752 756 21597394 10.1097/MNM.0b013e32834755df

